# Strangulated Hernia Reduced by a Cold Douche—Translation

**Published:** 1859-08

**Authors:** 


### ARTICLE VII.

[Translation by F. H. Evans, M. D., of Aberdeen, Mississippi.]

### *Strangulated Hernia Reduced by a Cold Douche.*

J. L. Petit tells an anecdote of an old woman who, in throwing a pail of cold water upon an irreducible hernia, overcame a strangulation in which all other means had failed. It is without doubt, not a very artistic proceeding; nevertheless there is in it a fact which should attract the attention of men of science, and which could put us on the way of a methodical application of cold affusions in the treatment of strangulated hernia. It is by this train of thought that M. Regnaud has been induced to employ as a substitute for a pail of water, a cold douche of great power.

C*** Jean Baptiste, laborer at port, age 29, was received into the Marine hospital the 28th of December, 1856, at 6, A. M.—he bore an inguinal hernia of the right side, dating back a year, and descending just into the scrotum. The patient had worn no truss, except for a few days after the accident which brought on the hernia, and since that time, he has been content to return it on going to bed at night, and resigned to the sight of it upon the slightest exertions at work. 27th---evening.---After an effort, he saw his hernia protrude more than usual, and he could not reduce it; the next day he presented himself at the hospital with all of the signs of an intestinal strangulation. 28th---(First of the strangulation.)---The tumor, which is larger than the fist of an adult, is firm, smooth, irreducible, and gives some pain under pressure. There is from time to time, hiccough and nausea. The attending surgeon made fruitless attempts at reduction by taxis, the patient being plunged into an anæsthetic sleep. C*** is put into a warm bath for an hour.

At 8. New attempts by taxis, without result. 20 leeches are applied upon the tumor, disposed in such a manner as to leave a large track for the incision, if an operation becomes necessary. A poultice is applied after the leeches, but the strangulation persists, and the general disturbance is aggravated; the expression of the face is uneasy; the hiccough more frequent, and vomiting has already set in.

At 2.30. Another bath of an hour and a quarter, and an injection of oleum ricini.

29th December.---Has passed a bad night; the hiccough has fatigued him very much; he has vomited at several times porraceous matter, having no particular odor; he has returned but little of the injection administered last evening.

At 6.30, A. M. Another warm bath, prolonged just to 8, A. M. On coming out of the bath, is placed upon his bed. He complains a great deal. The abdomen is sensitive to pressure. The tumor is painful to the touch, but it appears a little less voluminous than last night. This induced another attempt at the taxis. Under the second administration of chloroform, pushed just to muscular relaxation. This new, prolonged and energetic taxis produced no diminution of the tumor, that could give rise to a hope of the reduction of the intestine, and the patient is abandoned for several moments.

At 8.30, everything being ready for the operation, M. Regnaud decided to try as a last means, the cold douche. The reservoir of which is raised 60 feet above the place where the baths are administered. The patient is carried into a room reserved for that purpose. The temperature of the air is about 32F. A blanket is opened upon the floor, upon which the patient lies in the dorsal decubitus, his knees flexed, and his thighs separated, a cushion is placed under the pelvis so as to elevate this part, while the head itself is in a position much lower; the patient is made to hope that they are preparing to put him in a hot bath; a second blanket is thrown over his chest which warms him and hides from his sight the irrigating tube. The moment it is announced that he is prepared, a violent jet of cold water is thrown upon the scrotum from a distance of 15 inches. This jet is also cast by design, upon the abdomen, and produces a shock which almost takes the breath of the patient. From this first jet, which lasted but a few seconds, the tumor seemed to have diminished in volume.

As soon as the patient could breathe, another jet was ap. plied, and continued at a distance of 30 inches. The patient gave expression to the most horrible cries, and wished they would kill him. His face turned blue; four men held him with difficulty, while, with one hand, the surgeon threw the jet upon the scrotum and abdomen; he manipulated lightly the tumor with the other; he felt there a slight quivering; the subject is more agitated than ever; he lets escape from the anus a yellow oily fluid, the remainder of last night's injection. The tumor diminished sensibly, immediately the tube is abandoned, and a feeble pressure with both hands suffices to reduce the hernia, which sent out its characteristic gurgling. The operation lasted in all two minutes, and the patient enveloped in warm blankets is reported in the ward.

The 6th of January, he left the hospital with a firm truss.

[*L'Abeille Medicale*

